# Fasting Glucose of 6.1 mmol/L as a Possible Optimal Target for Type 2 Diabetic Patients with Insulin Glargine: A Randomized Clinical Trial

**DOI:** 10.1155/2021/5524313

**Published:** 2021-07-14

**Authors:** Lu Yuan, Fengfei Li, Yue Zhou, Rui Sun, Gu Gao, Qing Zhang, Yajuan Tang, Lu Dai, Jindan Wu, Jianhua Ma

**Affiliations:** ^1^Department of Endocrinology, Nanjing First Hospital, Nanjing Medical University, Nanjing, China; ^2^Department of Endocrinology, The First People's Hospital of Huaian, Huaian, China; ^3^Department of Endocrinology, The Affiliated Jiangning Hospital of Nanjing Medical University, Nanjing, China

## Abstract

To observe whether different insulin glargine titration algorithms based on fasting blood glucose (FBG) levels lead to different glycaemic variations (GVs) in type 2 diabetes (T2D) patients, a prospective, randomized, single-centre, comparative, three-arm parallel-group, open-label, treat-to-target, 24-week study was performed. A total of 71 uncontrolled T2D patients were recruited and randomized 1 : 3 : 3 into Groups 1, 2, and 3 (insulin titration goals of FBG ≤ 5.6, ≤6.1, and ≤7.0) for this study. The initiated insulin glargine dose was recommended at 0.2 U/kg/day and was then titrated following the FBG target. Patients were subjected to two 3-day continuous glucose monitoring (CGM) at baseline and the endpoint, wherein the CGM data were analysed, and the study's primary endpoint was the difference in 24 hrs mean amplitude of glycaemic excursion (MAGE) among the three groups. We observed that patients in the three groups had similar MAGE levels at the endpoint; however, Group 2 achieved a significant decrease in the MAGE level from baseline to the endpoint as compared to Groups 1 and 3 (all *p* < 0.05). We also observed that these patients had significant glycated haemoglobin A1c (HbA1c) value improvements as compared to the other two groups (all *p* < 0.05). Therefore, choosing an FBG level of 6.1 mmol/L as an insulin titration target provided significant GVs and HbA1c value improvements in T2D patients. Moreover, our data indicated that an FBG of 6.1 mmol/L could possibly be an insulin glargine titration target in T2D patients.

## 1. Introduction

Globally, China is among the top 10 countries for the highest number of diabetes patients (114 million), as well as total healthcare expenditure on diabetes [1]. Microvascular and macrovascular complication incidences, in particular, depend largely on the glycaemic control of diabetic patients [2]. Therefore, tasks for maintaining glucose control in these patients remain a focus of research.

A study conducted in China reported that 67.5% of Chinese type 2 diabetes (T2D) patients were on insulin therapy, but only 15% of them had glycated haemoglobin A1c (HbA1c) levels of less than 7.0% [3]. Among the blood glucose indices, HbA1c is a very useful parameter for reflecting 2–3 months of mean glucose control [4–6]. A previous study demonstrated that a reduction in HbA1c value in diabetes patients was associated with a decrease in the risk of microvascular and macrovascular complications [7]. However, it should be noted that HbA1c does not necessarily refer to daily glucose variations (GVs), since studies have found that patients with different GVs may have similar HbA1c values [8–10]. GV, more specifically, is a potential risk factor for oxidative stress, endothelial dysfunction, and inflammation, all contributing to vascular endothelial cell damage [11, 12]. Importantly, studies have already demonstrated positive correlations between GV and diabetic micro- and macrovascular complications [12, 13]. Therefore, the role of HbA1c and GVs should be highlighted in T2D to decrease diabetic complication incidences [8]. In response to this, continuous glucose monitoring (CGM), which continuously provides glucose readings at 5-minute intervals for several days, may be a potential tool for assessing GVs in T2D patients [14–16].

Insulin glargine (Lantus®) is a long-acting insulin analogue that provides the advantage of a once-daily dosage to maintain glycaemic control in T2D patients [17]. Interestingly, its initiation has been verified as a successful strategy for uncontrolled T2D that is unresolved even with 1-3 oral antidiabetic drugs (OADs) [18]. Furthermore, insulin glargine exhibits potential efficacy and safety in combination with different glucose-lowering agents. It causes significant improvements in HbA1c values [18] and low hypoglycaemia incidences [19].

In clinical practice, physicians often titrate the insulin glargine dosage according to the fasting blood glucose (FBG) level, since appropriate day-to-day blood glucose goals may play an important role in achieving glycaemic control in patients receiving insulin therapy [20]. It should also be noted that there was a wide variation between the FBG values to achieve the recommended HbA1c targets from various organizations. For example, FBG ≤ 5.6, 6.1, and 7.0 mmol/L were recommended as optional glucose control goals by the international and domestic guidelines [21, 22]. However, evidence of whether the optimization of insulin glargine titration targets is beneficial in GV of T2D patients is largely unknown.

Therefore, we performed a prospective study to determine whether different FBG targets lead to different GV values in the Chinese T2D population who were receiving insulin glargine analogue.

## 2. Materials and Methods

### 2.1. Study Design

This was a prospective, randomized, single-centre, comparative, three-arm parallel-group, open-label, treat-to-target study. The study protocol and patient consent forms were approved by the Institutional Ethical Committee of Nanjing First Hospital, Nanjing Medical University, and all procedures performed were in accordance with the ethical standards of Nanjing First Hospital and the Helsinki Declaration of 1964, as revised in 2013. Informed consent was obtained from all the patients for recruitment in the study, and this study was registered with ClinicalTrials.gov identifier: NCT02545842.

From October 2016 to April 2018, T2D patients who presented with insufficiently controlled diabetes for at least 3 months were enrolled as outpatients in the Department of Endocrinology, Nanjing First Hospital, Nanjing Medical University, China. The inclusion criteria were as follows: (1) willingness to participate in the study with a signed informed consent; (2) aged between 18 and 65 years; (3) T2D insufficiently controlled by 1–3 OADs with a stable dose for at least 3 months: (3a) if on 1 OAD, provided with the following doses (submaximum (half dose above) to maximum dose (for details, refer to the package inserts)) or the maximum tolerated dose allowed in the package insert, and (3b) if on 2–3 OADs, any dose was acceptable; (4) HbA1c value > 7% but ≤10.5%; (5) FBG level > 7 mmol/L (biochemistry result); (6) body mass index ≥ 20 kg/m^2^ but ≤40 kg/m^2^; (7) diabetes duration ≥ 1 year; (8) availability of physician prescription and patient's consent to start insulin glargine treatment; and (9) willing to undergo CGM. On the contrary, the exclusion criteria were as follows: (1) type 1 diabetes patients; (2) patients with acute diabetic complications (including unexplained severe hypoglycaemia in the last 6 months); (3) previous treatment with insulin for more than 1 month in the last 1 year, or treatment with insulin in the 3 months before the screening; (4) known hypoglycaemia unawareness or recurrent hypoglycaemia; (5) hypersensitivity to study drug or its excipients; (6) any clinically significant acute major organ or systemic disease, or any other situation which might be difficult for the 24 weeks follow-up, as judged by the investigator; (7) pregnant or breastfeeding women; (8) any mental disorders, lack of self-control, or inability to express accurately; and (9) involved in another clinical trial simultaneously or within 1 month before the start of the trial.

Following the inclusion of participants, the treatment code list was generated centrally, wherein treatments were allocated to each patient via a centralized system (interactive voice response system; IVRS/interactive web-response system; IWRS), and the randomization list was generated by a statistician who liaised with the IVRS/IWRS service. The patients were then randomized at a 1 : 3 : 3 ratio into one of the three groups: Group 1 (FBG level target ≤ 5.6 mmol/L), Group 2 (FBG level target ≤ 6.1 mmol/L), and Group 3 (FBG level target ≤ 7.0 mmol/L) at Visit 2 (week 0).

**Figure 1:**
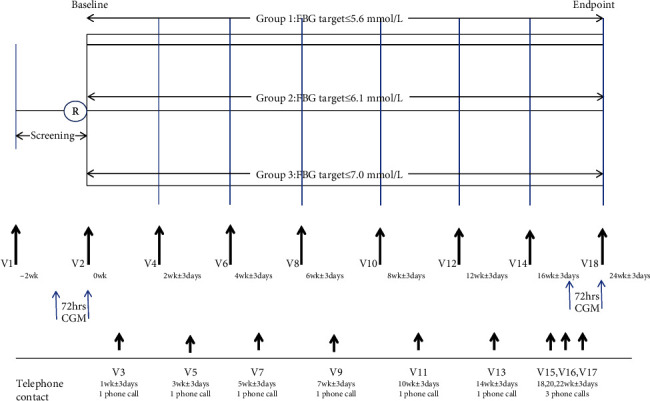
Study flow chart.

The study included a 0- to 2-week screening period and a 24-week treatment period, with 18 visits occurring as the study schedule. The study flowchart is further illustrated in detail in Figure 1. In brief, prior to the study, all patients were provided with instructions for measuring FBG levels and administering insulin glargine. The FBG level was recorded daily by patients before breakfast, and insulin glargine was self-administered once daily at bedtime (from 21:00 to 23:00) by subcutaneous injection into the abdomen (preferred route) using a SoloSTAR® disposable pen. Moreover, the initial insulin glargine dose was recommended at 0.2 U/kg/day, with a wave of 4 U, as permitted according to the patient's clinical condition by the investigator. The investigator would then titrate the insulin dosage according to the lowest value of the last three consecutive FBG values prior to each visit. The detailed insulin dose titration algorithm is presented in Table 1. Additionally, the insulin injection time and OADs taken by patients at baseline remained unchanged and were continued at a fixed and stable dose during the study.

### 2.2. Efficacy Measurements

#### 2.2.1. HbA1c

Fasting serum samples were collected and sent to the ICON Laboratory (Shanghai, China) for HbA1c measurements at Visits 1 and 18, respectively.

#### 2.2.2. Fasting Blood Glucose

Finger-striped blood glucose monitoring was carried out using the sponsor-provided glucose meter, which was performed by the recruited participants at home after at least 8 hours of fasting. Patients were instructed to report their FBG levels for at least 3 consecutive days of the visit week and at least 2 consecutive days of the visit-free week.

#### 2.2.3. CGM

All recruited patients were subjected to a two-time, 3-day, retrospective CGM (Sof-sensor, CGMS-Gold, Medtronic Incorporated, Northridge, USA) at 3 days of Visits 2 and 18, as described previously [23, 24]. During the two-time CGM period, patients were instructed to maintain moderate physical activity and have breakfast, lunch, and dinner at 07:00, 11:00, and 17:00, respectively, with a total daily caloric intake of 25 kcal/kg/day. The percentages of carbohydrates, proteins, and fats were 55%, 17%, and 28%, respectively. After the CGM data collection, the mean amplitude of glycaemic excursion (MAGE) was calculated manually for each patient by measuring the arithmetic mean of the ascending and descending excursions between consecutive peaks and nadirs for the same 24 hr period, wherein only absolute excursion values > 1 standard deviation (SD) were considered, as previously described [25, 26]. In addition, other glucose indicators, such as the 24 hr mean glucose concentration (MG), coefficient of variation (CV%), 24 hr standard deviation of the MG (SD), incremental area under the curve (AUC) of plasma glucose > 10.0 mmol/L, and the incremental area over the curve (AOC) < 3.9 mmol/L, were also recorded.

### 2.3. Outcomes

The primary outcome of this study was to identify whether different insulin glargine titration algorithms based on different FBG levels lead to different MAGE in T2D patients. The second outcome was the differences in HbA1c values, MG, SD, CV%, AUC values, and AOC values of the patients among the three groups.

### 2.4. Statistical Analysis

The analyses were performed using the SPSS 22.0 (SPSS, Science, Chicago, USA) statistical package. All variables were tested for the normal distribution of data and are presented as mean ± SD or as median (25^th^ percentile; 75^th^ percentile), based on the data's normal distribution. Parameters that did not fulfill a normal distribution were mathematically transformed to improve symmetry for subsequent analyses. One-way ANOVA, *T*-test, nonparametric tests, and chi-square test had been used for difference analysis among groups, respectively. All comparisons were 2-sided, and statistical significance for all analyses was set at *p* < 0.05.

## 3. Results

### 3.1. Demographic Characteristics

**Figure 2:**
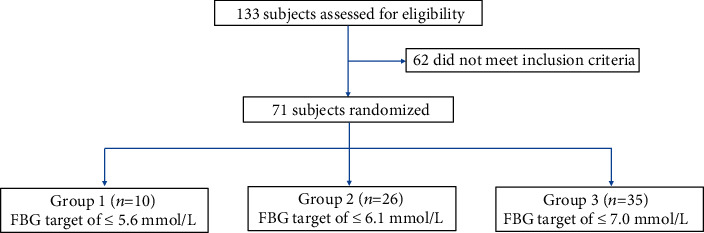
A total of 71 patients finished the study.

A total of 133 participants with T2D were assessed for eligibility, and 62 participants did not meet the inclusion criteria. Thus, the CGM data of 71 participants were collected and analysed at the endpoint (Figure 2).

There were no differences in the demographic characteristics of the recruited participants among the three groups (Table 2). The number of OADs in each group is further described in Table 2. There were no significant differences in the ratio of OADs among the three groups.

The FBG levels at baseline and the endpoint are shown in Table 3, wherein all patients among the three groups achieved significant improvements from baseline to endpoint (all *p* < 0.01). At baseline, FBG levels did not differ between the groups; however, the FBG levels in Groups 1 and 2 were significantly lower than those in Group 3 at the endpoint. After 24 weeks, 70.0%, 88.5%, and 80.0% of patients in Groups 1, 2, and 3, respectively, had FBG values within their predefined target range according to a preplanned titration strategy based on the lowest value of the last three consecutive FBG values.

To assess the effects of insulin glargine on glycaemic control in this study, we observed changes in HbA1c values from baseline to the endpoint. As Table 4 shows, all patients among the three groups achieved significant improvement in HbA1c values from baseline to endpoint (all *p* < 0.01). Importantly, the HbA1c level change percentage in Group 2 was significantly higher than that in Groups 1 and 3 (all *p* < 0.05). Furthermore, we observed that the percentage of patients with HbA1c levels less than 6.5% was numerically higher in Group 2 than in the other two groups (10.0%, 30.8%, and 17.1%, respectively), but the difference was not significantly different from either Group 1 or Group 3 (*p* > 0.05).

### 3.2. CGM Profiles

CGM data showed that the MG values of the recruited participants in all groups significantly improved upon comparing baseline to the endpoint values. In accordance with our HbA1c outcomes, our data showed that the MG value in Group 2 was significantly lower than that in Group 3 (*p* < 0.01) and was insignificantly reduced as compared to Group 1 (*p* > 0.05).

We then analysed the GV profiles of patients among the three groups. Our data indicated that patients had similar MAGE values at the endpoint, with the exception of patients in Group 2, showing a significant SD value reduction as compared to Group 3. However, we also observed that SD and MAGE value changes in Group 2 decreased from baseline to the endpoint as compared to both Groups 1 and 3 (*p* < 0.01, respectively) (Table 5).

Furthermore, CGM data showed that the hyperglycaemia (AUC values > 10 mmol/L) values were insignificantly decreased in Groups 1 and 3, and significant improvement from baseline to the endpoint was observed in Group 2. Although patients had similar hyperglycaemia values between Groups 1 and 3 at the endpoint, we observed a dramatic decrease in hyperglycaemia values at the endpoint in Group 2 as compared to Group 3 (Table 5).

### 3.3. Safety and Weight Gain

Patients in all three groups had the same hypoglycaemia values (finger-striped blood glucose < 3.9 mmol/L) or symptomatic hypoglycaemia incidences (0 (0, 1.25) vs. 0 (0, 1) vs. 0 (0, 0), *p* > 0.05). More importantly, our CGM data indicated that the hypoglycaemia (AOC < 3.9 mmol/L) values were similar either at baseline or the endpoint among the three groups (*p* > 0.05), showing no differences upon making comparisons between baseline and the endpoint, as well as among the three groups (*p* > 0.05) (Table 5).

**Figure 3:**
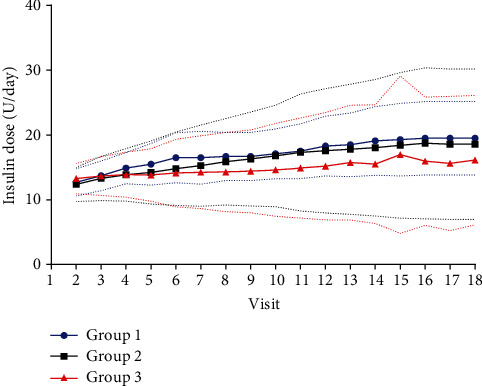
The insulin dose among the three groups during the study period.

Moreover, we observed that there was no difference in weight gain from baseline to endpoint among the three groups (*p* > 0.05). Insulin dose was also found to have had no difference among the three groups at the endpoint (Group 1: 0.3 ± 0.1, Group 2: 0.3 ± 0.1, and Group 3: 0.2 ± 0.1 U/kg, *p* > 0.05), as well as at each visit (Figure 3).

## 4. Discussion

This prospective study showed that T2D patients receiving insulin glargine therapy had day-to-day FBG values of less than 6.1 mmol/L, leading to a significant improvement in short-term and long-term glycaemic control. Therefore, the insulin glargine titration target threshold may be an FBG of 6.1 mmol/L in T2D patients, as indicated in our data.

In this study, not all patients achieved the preset FBG goals as assigned, with the ratio between the three groups being similar at 24 weeks. Specifically, the ratio in the BEYOND III was 70.1%, 67.6%, and 79.0% in Groups 1, 2, and 3, respectively [27]. Although most previous studies have employed HbA1c as the primary glycaemic control measure [28], this only provides an approximate measurement of glucose control, since it does not address acute/short-term GV or hypoglycaemic events [29]. CGM, on the contrary, is a useful tool that helps clinicians and diabetic patients to overcome the limitations of HbA1c in diabetes management [29]. Notably, many CGM parameters were found to reflect GV, such as MAGE, SD, and CV%. As mentioned previously, GV is a risk factor that contributes to the pathogenesis of diabetes micro- and macrovascular complications [30–32]. Additionally, strict FBG level control using insulin glargine may not lead to further GV improvement T2D in patients [33]. In this prospective study, patients with an FBG level of 5.6 mmol/L as the insulin glargine titration target failed to achieve the largest GV reduction. In contrast, our data showed that T2D patients in Group 2 had insignificant MAGE and SD decreases from baseline to the endpoint, while the MAGE and SD values in those of the other two groups were insignificantly increased. More importantly, we observed a significantly increased change amplification in MAGE and SD values from baseline to the endpoint in Group 2 as compared to either Group 1 or Group 3. However, despite these findings, the mechanism behind the different MAGE and SD change patterns among the three groups was unclear. We consider the reason might be that while hypoglycaemia (AOC < 3.9 mmol/L) values were similar either at baseline or the endpoint among the three groups, hyperglycaemia (AUC >10 mmol/L) was found to be significantly declined in Group 2 from baseline to the endpoint. Thus, glycaemia control in Group 2 was more stable than in the other two groups. Furthermore, although MAGE and SD values in Group 2 decreased significantly from baseline to the endpoint, CV% was conversely increased, which was possibly due to the dramatically decreased MG from baseline to the endpoint.

A 16-week prospective study conducted in China reported that T2D patients receiving insulin glargine and targeting an FBG of 6.1 mmol/L had nearly a 1.8 mmol/L reduction in MAGE values [34]. In agreement with the previous study, we also used an FBG of 6.1 mmol/L as the insulin titration threshold target, finding a 1.4 mmol/L improvement in the MAGE value (6.2 ± 3.2 to 4.7 ± 2.8 mmol/L) from baseline to the endpoint. However, we did not observe the same change trend in the other two groups, which used FBG levels of 5.6 and 7.0 mmol/L, respectively. As such, we have addressed this as another limitation of this study since we have no data backing up the underlying mechanisms.

Based on previous studies, MAGE was considered as the gold standard to reflect GV [35], while SD is simple and sensitive in reflecting GV [36]. MAGE, in particular, has contributed significantly to decreasing antioxidation capacity as compared to chronic sustained hyperglycaemia in patients with different types of glucose regulation [37]. In fact, CGM-based MAGE has been significantly correlated with urinary 8-iso-prostaglandin F2a levels, IMT, and Gensini score in T2D patients [30, 38, 39]. In relation to this, we further observed that patients receiving insulin glargine with an FBG of 6.1 mmol/L as titration threshold exhibited an SD change from 2.3 ± 1.0 to 1.8 ± 0.9 mmol/L during the 24-week treatment. However, the MAGE and SD values in T2D patients at the endpoint in this study were higher as compared to the recommended values in the Chinese population [40]. Therefore, studies on insulin glargine aimed at reducing MAGE and SD values are warranted in the future.

A previous study reported that T2D patients receiving OADs with add-on glargine therapy for 16 weeks had their HbA1c levels reduced from 8.35 ± 0.24 to 7.14 ± 0.16% [34]. Similarly, in our study, we observed that T2D patients receiving insulin glargine had a significant reduction in HbA1c value after the 24-week treatment period, with HbA1c values varying from 6.8 ± 0.7 to 7.3 ± 0.8% at the endpoint. Interestingly, in accordance with our outcome regarding the benefits of GV improvement in patients with an FBG of 6.1 mmol/L as the titration target, we also found that patients in Group 2 had a significant HbA1c value reduction as compared to those in Groups 1 and 3, which was in agreement with a previous study reporting that using an FBG of 6.1 mmol/L as the insulin glargine titration target led to significant HbA1c value improvements in T2D patients [27].

It is logical to see a significant difference in insulin doses among the three groups, due to their varying FBG targets. However, we did not observe any differences among the three groups at Visit 2 and at the endpoint, even during the insulin titration period (from Visit 3 to Visit 17). Another strength of this study was that all recruited participants received a mean insulin dose of 0.3 U/kg at the endpoint among the three groups, which may have additionally benefited glycaemic control. This explanation was observed in a study which showed that the relationship between high basal insulin doses and glycaemic control was nonlinear, with increasing insulin doses leading to smaller FBG and HbA1c reductions for doses > 0.3 U/kg/day and a plateauing effect at 0.5 U/kg/day [41].

Moreover, hypoglycaemia and weight gain [42] were closely associated with insulin therapy in T2D patients, in which the lower FBG target may lead to an insulin dose increase in T2D patients, which was a risk factor for hypoglycaemia and weight gain. However, another study indicated that treatment to target an FBG < 5.3 mmol/L with insulin glargine was not associated with significantly increased risk for hypoglycaemia as compared to a target of FBG < 6.1 mmol/L [33]. We also found that patients among the three groups with insulin glargine therapy lasting for 24 weeks had similar hypoglycaemia incidences. Additionally, our data demonstrated that all recruited patients receiving insulin glargine had similar weight gain at the endpoint. Thus, insulin glargine therapy is a safe and effective treatment for T2D patients, even with strict FBG targets.

## 5. Conclusions

In conclusion, an FBG level of 6.1 mmol/L as the insulin titration goal provided significant improvement in GV and HbA1c values in T2D patients. Therefore, an FBG of 6.1 mmol/L may possibly be a viable threshold for insulin glargine titration targeting in T2D patients.

## Figures and Tables

**Table 1:** Titration regimes for recruited subjects.

FBG (mmol/L)	Insulin dose
All groups < 3.9 or nocturnal hypoglycaemia	-2 U

Group 2: 3.9 < FBG ≤ 5.6	According to clinical condition by the investigator -1-2 U or no change
Group 3: 3.9 < FBG ≤ 6.1	

Group 1: 3.9 < FBG ≤ 5.6	No change
Group 2: 5.6 < FBG ≤ 6.1	
Group 3: 6.1 < FBG ≤ 7.0	

Group 1: 5.6 < FBG < 10.0	+2 U
Group 2: 6.1 < FBG < 10.0	
Group 3: 7.0 < FBG < 10.0	

All groups > 10.0	According to clinical condition by the investigator +2-4 U

FBG: fasting blood glucose.

**Table 2:** The baseline characteristics of subjects among the three groups.

Group	1	2	3	*p* value
Gender (M/F)	5/5	14/12	26/9	0.16
Age (year)	56.5 ± 8.9	57.9 ± 4.7	53.8 ± 7.3	0.06
Duration (month)	103.1 ± 66.4	113.6 ± 62.8	107.9 ± 65.1	0.89
Numbers of OADs (*n*)				0.06
1	1	8	2	
2	8	12	24	
3	1	6	9	
OADs				
Met(-/+)	4/6	7/19	4/31	0.10
SU (-/+)	2/8	9/17	5/30	0.17
Weight (kg)	68.8 ± 10.2	65.2 ± 12.6	70.9 ± 9.5	0.13
BMI (kg/m^2^)	25.7 ± 3.1	24.6 ± 3.0	25.6 ± 2.4	0.29
Waist (cm)	92.2 ± 10.6	89.9 ± 9.8	93.0 ± 7.6	0.41
Hb (g)	139.4 ± 10.5	143.6 ± 10.0	144.0 ± 11.3	0.48
Cr (mmol/L)	62.5 ± 13.0	59.0 ± 14.7	65.0 ± 12.2	0.26
ALT (U/L)	31.1 ± 15.9	29.3 ± 14.5	35.6 ± 18.3	0.34
AST (U/L)	20.0 ± 6.5	20.8 ± 7.2	21.3 ± 10.2	0.91
TG (mmol/L)	2.1 ± 1.6	2.0 ± 1.4	2.0 ± 1.1	0.94
TC (mmol/L)	5.2 ± 1.0	5.5 ± 1.2	5.2 ± 1.1	0.59
HDL (mmol/L)	1.3 ± 0.3	1.5 ± 0.3	1.3 ± 0.3	0.09
LDL (mmol/L)	2.8 ± 0.6	2.9 ± 0.9	2.8 ± 0.9	0.98

BMI: body mass index; Hb: hemoglobin; Cr: creatinine; ALT: alanine aminotransferase; AST: aspartate aminotransferase; HDL-c: high-density lipoprotein cholesterol; LDL-c: low-density lipoprotein cholesterol; TG: triglyceride; TC: total cholesterol; OADs: oral antidiabetic drugs; Met: metformin; SU: sulfonylurea.

**Table 3:** The FBG among the three groups.

Group	V 1	V 18	*p*
1	8.3 ± 0.9	5.5 ± 0.5	0.00
2	8.6 ± 1.9	5.4 ± 0.6	0.00
3	8.3 ± 1.6	6.4 ± 1.1^∗^^#^	0.00

∗: vs. Group 1 *p* < 0.05; #: vs. Group 2 *p* < 0.05; FBG: fasting blood glucose.

**Table 4:** The changes of HbA1c among the three groups.

Group	V 1	V 18	*p*	The absolute change value	The percent of change value
1	8.0 ± 0.7	7.2 ± 0.6	0.00	0.8 ± 0.3^∗^	9.7 ± 3.4^∗^
2	8.4 ± 0.8	6.8 ± 0.7	0.00	1.6 ± 0.9	18.5 ± 9.3
3	8.5 ± 0.8	7.3 ± 0.8^∗^	0.00	1.2 ± 0.8	13.8 ± 9.2^∗^

∗: vs. Group 2 *p* < 0.05; HbA1c: glycated hemoglobin A1c (%).

**Table 5:** The CGM profile of the three groups.

Group	1	2	3	*p*
MG				
Baseline	10.1 ± 1.7	10.4 ± 2.0	10.0 ± 1.7	0.70
Endpoint	8.2 ± 1.3^∗^	7.4 ± 1.2^∗^	8.9 ± 1.8^∗^	0.00
*Δ*	1.8 ± 2.0	3.0 ± 1.9	1.1 ± 2.1	0.00
SD				
Baseline	1.8 ± 0.8	2.3 ± 1.0	2.1 ± 0.8	0.30
Endpoint	2.3 ± 1.0	1.8 ± 0.9	2.6 ± 1.0^∗^	0.01
*Δ*	−0.5 ± 1.2	0.5 ± 1.4	−0.4 ± 1.2	0.01
CV%				
Baseline	17.8 ± 4.5	22.1 ± 8.9	21.7 ± 8.4	0.35
Endpoint	27.7 ± 9.7^∗^	24.1 ± 10.5	28.7 ± 10.2^∗^	0.23
*Δ*	−10.0 ± 11.3	−2.1 ± 14.6	−7.0 ± 11.6	0.17
MAGE				
Baseline	4.4 ± 1.3	6.2 ± 3.2	5.5 ± 2.7	0.22
Endpoint	5.7 ± 3.2	4.7 ± 2.8	6.0 ± 2.8	0.26
*Δ*	−1.3 ± 2.9	1.4 ± 3.8	−0.5 ± 3.3	0.04
AUC				
Baseline	183.3 (60.7, 441.7)	327.2 (115.4, 694.6)	212.6 (64.8, 502.9)	0.63
Endpoint	104.1 (17.7, 204.1)	19.7 (0, 120.0)^∗^	128.8 (31.1, 401.0)	0.02
*Δ*	102.7 (-45.7, 282.5)	229.4 (98.6, 502.7)	26.41 (-110.3, 349.9)	0.01
AOC				
Baseline	0 (0, 0)	0 (0, 0)	0 (0, 0)	0.58
Endpoint	0 (0, 0)	0 (0, 0)	0 (0, 0)	0.44
*Δ*	0 (0, 0)	0 (0, 0)	0 (0, 0)	0.49

*Δ*: baseline value-endpoint value; ∗: baseline vs. endpoint *p* < 0.05; MG: the 24 hrs mean glucose concentration (mmol/L); SD: the 24 hrs standard deviation of the MG (mmol/L); CV%: coefficient of variation (%); MAGE: the 24 hrs mean amplitude of glycaemic excursion (mmol/L); AUC: the incremental area under the curve > 10 mmol/L (mmol/L∗day); AOC: the incremental area over the curve < 3.9 mmol/L (mmol/L∗day).

## Data Availability

The data sets generated during and/or analysed during the current study are not publicly available but are available from the corresponding author on reasonable request.
